# Simulated learning interventions to improve communication and practice with deaf and hard of hearing patients: a systematic review and qualitative synthesis

**DOI:** 10.1007/s10459-025-10452-5

**Published:** 2025-07-09

**Authors:** Julia Terry, Rachel Wilks, Joanne Davies

**Affiliations:** Swansea, Wales UK

**Keywords:** Simulation-based-education, Immersive learning, Virtual reality, Deaf, Hard of hearing, Empathy, Communication

## Abstract

Virtual and simulated patients are increasingly used in health professional education as learning about patient needs in a safe space greatly benefits student knowledge and skills and increases their empathy towards patients. Yet to date there has been limited focus on using simulated learning techniques in health professional education to promote learning about D/deaf and hard of hearing patients. We used systematic review methodology to search, identify, appraise and abstract relevant articles across CINAHL, MEDLINE, ASSIA and Proquest Central, Scopus, Web of Science and Cochrane databases yielding a total of 1112 papers. After removing duplicates, inclusion criteria were defined and applied, resulting in 132 articles retrieved for full-text review. Six articles met all inclusion criteria, addressing simulated learning methods for health professional students that provide educational opportunities about Deaf patient experiences. Findings suggest that a myriad of possible simulation modalities can be developed that include opportunities to learn about the D/deaf patient experience and to consider learning about communication and application of knowledge to a specific topic environment. This qualitative synthesis provides insight into potential methods and styles of delivery, whilst noting a very small number of studies in this area. Future research should focus on rigorous and longitudinal studies to understand more about student learning and how interventions impact on their communication and encounters with D/deaf patients.

**Supplementary Information:**

The online version contains supplementary material available at 10.1007/s10459-025-10452-5.

## Terms

Throughout our protocol and subsequent work, Deaf with a capital D, refers to people who identify as, and are culturally Deaf and may be Sign language users; and deaf people with hearing loss are referred to with a lower-case d for deaf (usually not signers). In this paper we use the terms Deaf, deaf, d/Deaf (as an inclusive term), and hard of hearing.

## Introduction

The healthcare experiences of D/deaf and hard of hearing patients are fraught with unique challenges that often stem from a lack of understanding and accommodation within the healthcare system resulting in significant barriers for Deaf and hard of hearing people accessing healthcare services (Abou-Abdallah & Lamyman, 2021; James et al., 2022; Rezende et al., 2021; Terry et al., 2024). The multifaceted challenges D/deaf individuals face are consistently highlighted in the literature, and relate to health behaviours and literacy (Almusawi et al., 2021; Payami, 2021); barriers in communication (Nicodemus et al., 2020; Yet et al., 2022); systemic issues in terms of access (Foltz & Shank, 2020; Fuentes-López, 2020), and poor doctor-patient relationships (Pereira et al., 2020; Rannefeld et al., 2023).

Communication is a central challenge in the healthcare experiences of D/deaf and hard of hearing individuals. Effective communication is vital for ensuring that D/deaf individuals can make informed health decisions (Anderson et al., 2020). The limitations of written communication and the inconsistent availability of qualified sign language interpreters pose significant difficulties. According to Grote and O’Brien (Grote et al., 2021), these barriers often force D/deaf patients to rely on inadequate communication methods, such as lip-reading or using family members or friends to act as interpreters, compromising the accuracy and confidentiality of health information exchanged. Furthermore, D/deaf and hard of hearing patients often face difficulty in scheduling appointments that accommodate their communication needs to the lack of accessible medical forms and signage (Lee et al., 2021; Schniedewind et al., 2021), as well as further physical and administrative barriers that hinder their access to timely and appropriate healthcare services. These challenges are compounded by healthcare providers’ limited awareness of Deaf culture and the accommodations necessary to provide inclusive care. Another significant barrier is the variability in healthcare providers’ awareness and understanding of Deaf culture and communication needs (Jacob et al., 2022; Terry et al., 2024). Many healthcare professionals are not trained in a Signed Language and may not be aware of the importance of using qualified interpreters. This lack of training can result in ineffective communication strategies, such as assuming that speaking slowly or loudly will help or attempting to communicate through lip-reading. Additionally, a lack of professional interpreter services also leads to longer appointment times and delays in receiving care, further exacerbating health inequalities (Terry et al., 2021).

Simulated learning and virtual reality (VR) simulations are emerging as innovative educational tools to enhance healthcare professionals’ understanding of working with certain patients (Shin, 2018) as well as providing experiential learning opportunities that bridge theoretical knowledge and practical skills (Kim & Kim, 2023). Educational interventions using simulated learning have demonstrated significant benefits, such as positive attitude changes among participants from sympathy to empathy and advocacy for disability rights (Havercamp et al., 2021; McKenney, 2018), as well as promoting acceptance and understanding of peers with disabilities (VanPuymbrouck et al., 2020). Simulated learning in health professional education programmes has the potential to transform attitudes and enhance professional competence by providing immersive experiences that simulate real-world interactions with D/deaf and hard of hearing individuals, improve communication skills, and gain insights into Deaf culture and communication preferences.

Utilising virtual simulations, VR, and 360-degree videos, these methods immerse healthcare professionals/students in scenarios that replicate real-world challenges (Halley & Connelly, 2022). Studies have shown simulation of clinical interactions (Yuksel & Unver, 2016) where providers must navigate the absence of sound, relying solely on visual cues and sign language interpreters, and also allow for repeated practice in a controlled environment improving proficiency and confidence in using Signed Language and interpreters (Li et al., 2017). Moreover, simulated learning can enhance Deaf awareness by illustrating the cultural and linguistic preferences of the Deaf community (McCartney et al., 2023). Simulated learning approaches can ensure healthcare professionals not only improve their technical skills but also develop a respectful and inclusive attitude toward Deaf and hard of hearing patients. By simulating the communication barriers and healthcare challenges faced by Deaf individuals, healthcare providers will gain a greater understanding and sensitivity, thus playing a crucial role in preparing professionals to work effectively with D/deaf and hard of hearing patients. To the best of our knowledge, there has been no systematic review of the use of virtual or simulated D/deaf patients in health disciplines.

Our research question was: ‘What is the available evidence regarding health professional training programs using virtual reality, technology, or simulation approaches to educate about D/deaf and hard of hearing patients’ experiences?’ The aims of this systematic current review were to:

1. Provide an overview of the approaches used to teach about Deaf patient experiences using simulated learning/virtual reality approaches and techniques
2. Examine and compare learning outcomes and findings across different programmes

## Methods

### Design

The methodology for this systematic review was based on the Preferred Reporting Items for Systematic Reviews and Meta-Analyses Protocols (PRISMA-P) (Moher et al., 2015; Page et al., 2021) and was reviewed and revised by the project steering group. PRISMA methodology 2020 was chosen to synthesize diverse study designs, including both RCTs and qualitative studies, as PRISMA’s applicability and flexibility in systematic review processes is a valuable tool to assist evaluation of studies that evaluate the effects of health interventions, irrespective of the design of the included studies (Page et al., 2021). The review protocol was published on Open Science Framework- https://osf.io/rxwdp/. The PRISMA checklist is presented as an online supplement table, along with searches from all databases (Medline, ASSIA, Proquest Central, Cinahl, Scopus and Web of Science).

### Search strategy

We conducted a planned literature search from September to November 2023, revisiting again in July 2024, over the following seven electronic databases which cover health, care and education including simulation-based education: CINAHL, MEDLINE, ASSIA and Proquest Central, Scopus, Web of Science and Cochrane database of systematic reviews and Cochrane trials. The general search terms included those related to deafness, sign language, virtual reality, technology, simulation-based education and learning, knowledge and understanding. We used the following search terms: (deaf* OR hard of hearing OR hearing impaired OR d/hh OR d/Deaf) AND (sign* language OR British Sign Language OR BSL) AND (virtual reality OR VR OR 3D technology OR simulat*) AND (empath* OR knowledge OR awareness OR perspective OR experience* OR understand*). In addition, the search was limited to original articles that were peer-reviewed and written in English. Publication date and period were not restricted, and all relevant studies were included. Finally, a manual search of reference lists was conducted from previous review articles. The study was exempt from ethical approval as it was a literature review study.

### Inclusion and exclusion criteria

Inclusion criteria: published research articles and dissertations, literature reviews and PhD theses specific to:

- A focus on teaching/training people with the aim to develop the awareness/knowledge of others so they learn about Deaf people’s experiences
- Programs or training where the focus is on learning about Deaf or hard of hearing people or people using Sign Language
- Programs or training where the focus is on training using computer-based learning, Virtual reality/immersive technologies or simulation-based educational modalities.

Exclusion criteria: papers that were not empirical studies, papers without a focus on learning about Deaf or hard of hearing people, or deaf awareness, papers without a focus on virtual reality/ immersive technology or simulation-based education modalities in terms of learning.

There was no restriction on the types of studies included. Randomised Controlled Trials, case studies, experimental, cross-sectional, and qualitative studies were all considered where the inclusion criteria were met.

Human Ethics and Consent to Participate declarations: not applicable, as this project was a literature review.

### Data extraction, data synthesis and assessment processes

After retrieving the search results from different databases, duplicates were removed and remaining results uploaded to Rayyan (Ouzzani et al., 2016). The initial screening and reading of abstracts of search results was done independently by two members of the research team (JT and RW). Rayyan software is a research collaboration platform that enables researchers to complete literature reviews and systematic reviews. Having first agreed on the review’s inclusion criteria, researchers then independently click ‘yes’, ‘no’ or ‘maybe’ buttons. In cases of uncertainty, ‘maybe’ articles were marked for discussion and independently screened by both researchers, with disagreements resolved through meetings and inspections of articles against inclusion/exclusion criteria and weighing up the articles’ significance until an agreed decision was reached. The selected full texts were read, and the following information was collected from each article: basic information (article name, authors, year of publication, country), study aim, sample, study description and outcomes.

Two reviewers independently read the extracted papers in line with the study’s inclusion criteria which were papers that focused on training programmes to increase awareness and knowledge about Deaf and hard of hearing individuals—particularly those who use sign language—through educational approaches including in-person instruction, computer-based learning, and immersive technologies such as virtual reality and simulation. Both authors critically examined the extracted data and categorized the validity evidence, bearing in mind their own positionalities and potential biases throughout the selection and analysis process. The first reviewer is an experienced academic, is hearing, and has been learning Sign Language, and teaching health professional students for twenty years. The second reviewer is a Deaf researcher and is a native Sign language user. Disagreements were resolved through discussion until consensus was achieved. All authors contributed to data analysis and synthesis and identified evidence gaps. This was achieved by summarizing the literature, initially for distillation with the project steering group. Findings assisted phase two of the Deaf SUSIM project, and informed the development of the protocol to develop three modules of learning which included an immersive VR prototype (primary care clinic experience with a focus on awareness and empathy training), and evaluative review with Deaf experts, students and lay members to assess product quality.

## Results

**Fig. 1:**
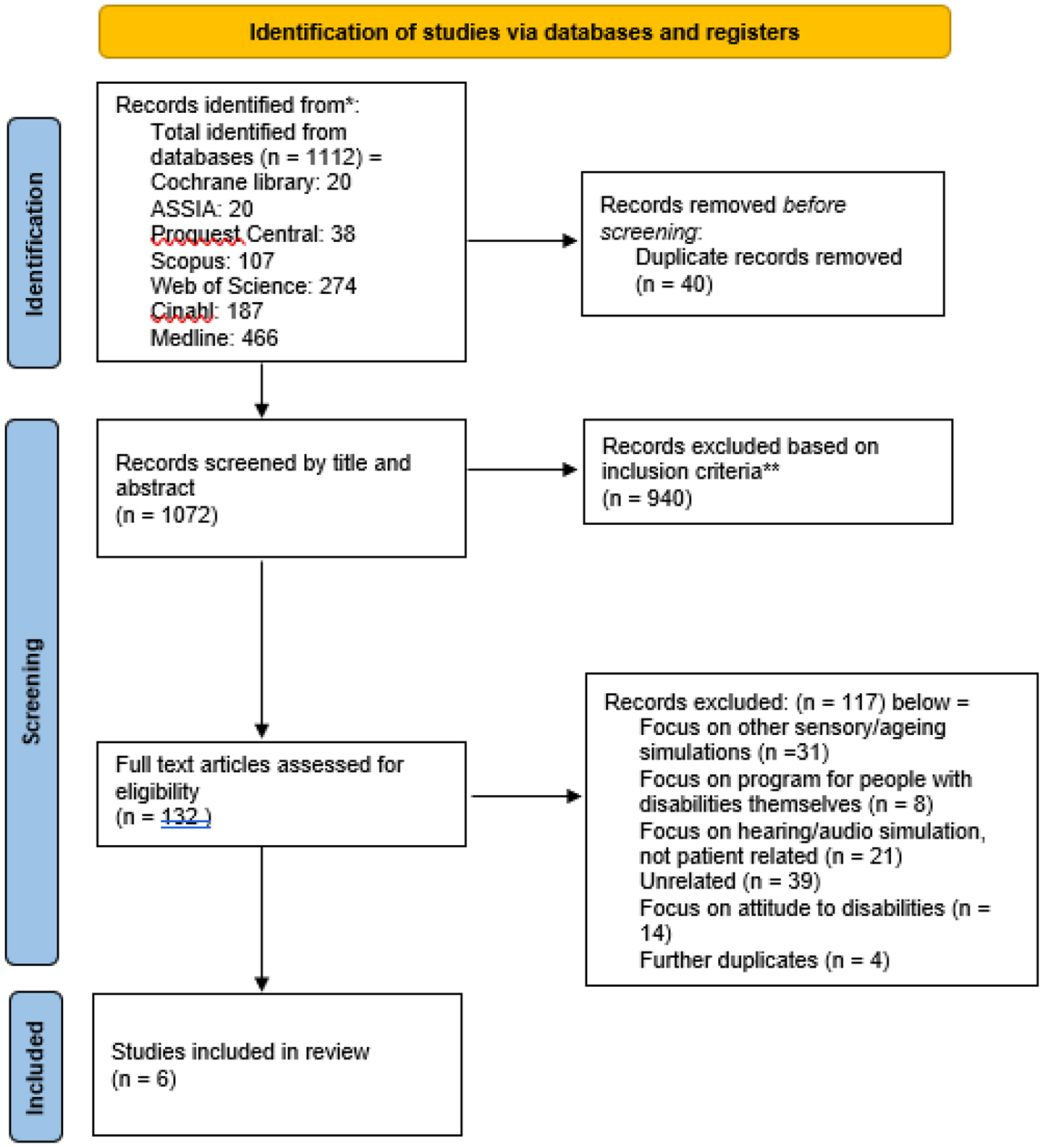
PRISMA diagram showing process of systematic review to identify and screen papers

A total of 1112 records were identified. After removal of duplicates (n = 40) and exclusion of irrelevant references (n = 940), 132 studies remained for full-text screening. Of these, 6 studies fulfilled the inclusion criteria (see flowchart in Fig. 1).

Table 1 shows a summary of all the included articles, with all six articles published between 2008 and 2023, with the majority published in the last four years. The included studies included four from USA, one from Turkey and one from UK. Two of the studies focused on nursing students (n = 2), with the others focusing on medical students (n = 1), dental students (n = 1), nurse practitioners (n = 1), and research staff in a health facility (n = 1). Sample sizes varied from five (Anderson et al., 2020) to 44 (Sanders et al., 2008), with the majority having a sample of under 25 participants (n = 4), with one not including sample information (Halley & Connelly, 2022). The results of this review are structured under two broad themes: characteristics of simulation intervention and study outcomes, in order to summarise relevant findings.

**Table 1:** Summary of included studies of simulation with Deaf patient focus

Source	Study aims	Sample	Type of simulation modality	Study description	Outcomes
1. Anderson et al. (2020), USA	To promote Deaf engagement in research about Deaf communities; increase the number of Deaf individuals who participate in general population biomedical research; and generalise findings to improve research accessibility for general population Objective of this Phase three study was to examine feasibility and acceptance of researcher training intervention in terms of recruitment, retention, engagement, participant satisfaction, intervention fidelity and feasibility of assessment procedures	Five research staff	Simulated environment with Standardised patient	Three-phase community engagement approach to adapt informed consent process and to train research staff. Following community forums and focus groups in phases one and two, a 4-hour study session including simulation was piloted on five staff involved in informed consent for human subjects’ research ‘Sign Here: how to conduct informed consent with Deaf research participants’	All participants completed a pre-intervention evaluation including experience of working with Deaf communities and self-assessment of perceived informed consent skills. Following Simulation #1, team members rated participant performance, with suggestions for improvement. Training videos were then observed, and participants self-rated their performance, noting areas for improvement. Following Simulation #2 team members again rated participant performance, and participants self-rated All five participants strongly agreed the study video and the simulation-based practice was helpful, with 4/5 rating they ‘strongly agreed’ that they felt more confident delivering culturally appropriate informed consent following the simulation. Participants with ‘very little’ experience with Deaf communities made greater gains in post intervention performance Appropriate audience for intervention is hearing research staff with little experience of Deaf communities
2. Blok et al. (2023), USA	To describe the processes of development of a 360-video education intervention using a user-centred design approach with a three-step process	22 students on nurse practitioner course used the headsets	Virtual reality: 360 video	Followed a user- centred design approach with three overall steps of defining user needs, conducting iterative prototyping and testing the intervention with target users 1. User needs were identified through focus groups with Deaf people; 2. educational and 360-video resources were created based on collected data; 3. Feasibility and usability testing is described about a series of educational videos and virtual reality in the classroom setting for students to understand and empathize with the deaf and hard-of-hearing (HOH) population’s challenges with the healthcare system	Focus group participants reported multiple areas of concern that Deaf populations experience, which were then included in video scripts. Delivery in classroom setting was deemed feasible with good usability System Usability Survey total scoring was 77.5, where 68 or above is considered above average Noted that technical preparation is needed for some students Anecdotal evidence from participants identified the importance of using this educational intervention Noted that 360 films could be less costly, but a VR experience would be more immersive with a headset
3. Halley and Connelly (2022), USA	To explore nursing students’ experiences of simulated health care appointments with deaf standardized patients, with virtual appointments mediated by student sign language interpreters	Undergraduate nursing students and undergraduate American Sign Language (ASL) interpreting students (n = not stated)	Virtual simulation experience with standardised patients	Faculty, students and Deaf community partners met on Zoom to conduct simulated health appointments with hearing nursing students and Deaf standardised patients Prior to meeting, nursing students and interpreting students were informed they would lead consultations with deaf patients about a variety of health conditions (e.g. hypertension, diabetes and osteoporosis). Nursing students led the simulated appointment with interpreting students instructed to interpret, with graduate interpreting mentors monitoring and Faculty staff observing. All parties then reconvened for de-briefing and feedback. Focus of de-briefing was a reflective discussion promoting self-reflection and critique using Gather, Analyse and Summarise (GAS) (Abulebda et al., 2020) Focus was preparing professionals to collaborate in face to face and virtual environments	Both nursing and interpreting students reported increased confidence in working as part of a healthcare team. Agreed the experience prepared them for future work. Nursing students reported learning first-hand how to collaborate with interpreters as part of the healthcare team Nursing students reported important lessons such as how to adjust and increase appointment time needed, and that they acquired basic ASL greetings signs Nursing students reported an increase in their clinical judgement skills, as they needed to respond to questions from the individual standardised patients, and the experience required them to think critically and creatively. All students reported the experience was positive in strengthening their professional identities
4. McLaughlin et al. (McLaughlin et al., 2020), UK	To gain an understanding of medical students’ experiences of being placed in the role of a Deaf or hard of hearing patient using a virtual reality (VR) simulation	A convenience sample of ten medical students were sought and obtained	VR with hearing loss simulation	Following the production of a VR learning tool about a Deaf patient visiting a GP with a ‘upper respiratory tract infection’. Scripting and storyboarding developed first a scenario showing poor communication from the GP and second, a scenario showing effective communication. Crucial to post-production was manipulation of recorded sounds to simulate a realistic auditory recreation of deafness. A user briefing for the VR experience was developed One to one interviews were conducted with each participant, who were asked to draw a ‘Rich picture’ as an elicitation tool that symbolised their VR experience	Analysis of interviews resulted in identification of four main themes: 1. ‘much more than watching a video’ 2. ‘hearing through their ears’ 3. ‘not just what you can’t hear ... but how it makes you feel’ 4. Redirecting my future professional self VR has potential to provide a novel complementary training method for medical students
5. Sanders et al. (2008), USA	To explore pre and post comfort and knowledge scores of dental students engaging with a virtual patient programme, where virtual patient has developmental disabilities and sensory impairment	44 third – year dental students (out of 52) received the module	Multi-media virtual patient (CD-ROM)	Two training modules featuring virtual patients were developed for dental students, the second module featured ‘Daniel’ who displayed deafblindness and a painful molar, as the clinical presentation, as a new patient at the dental surgery The purpose was to increase student comfort levels and knowledge of patients with significant sensory impairment. The module simulated a patient encounter using visual observations and clinical decision-making. Content developed by team of ten including those with lived experience and design specialists Used a pre and post knowledge test to measure student comfort and knowledge levels	Student level of comfort (i.e. perceived difficulty), and knowledge were measured pre and post module. Student comfort was measured using the Disability Situations Inventory, and knowledge was measured using multiple choices items based on the module content. Participants were also asked to rate the need for the programme, along with ease of use and navigation 38/44 participants completed the comfort measure, with significant improvement in comfort levels shown. 39/44 participants completed the knowledge measure with knowledge gains shown as statistically significant. Two students did not complete due to technical difficulties, and a further three cited no reason for failing to complete the module. 40/44 participants completed the usability scale, with students agreeing on a need for the programme Researchers note that more work is needed to see if knowledge and attitude change are reflected in actual patient care
6. Yuksel and Unver (2016), Turkey	To develop a pathway for nursing students to use when communicating with deaf patients	22 senior nursing students placed as interns in a university hospital in Turkey	Standardised patient method with simulations performed and recorded three times	A simulated patient, where students either took role of being the Deaf patient or the nurse, had to imagine their roles and read script. They were instructed to show empathy and be aware of their thoughts and actions Recordings of the simulated interactions were then shown to the rest of the cohort to analyse and reflect on content. After each reflection, a further simulation was held (three in total) with adjustments made each time in line with clinical decision making Recorded debriefing sessions were transcribed and themes identified as: patient preferences, sitting position during training, speech speed and content, body language, eye contact and preparing a pathway	Noted that the educational experience would be improved if a wider range of students were involved (not one role playing the nurse, and a further three in the patient role). A learning opportunity that involves all students would be preferable Nursing students reported difficulty in communicating with a patient with disabilities due to their limited experience. With further iterations of the simulation, improvements in communication were noted due to increased practice A sign language guide was recommended to facilitate communication with deaf patients in health care settings

### Characteristics of simulation intervention

The characteristics of simulation intervention in the included studies refers to the type of simulation method used, and the development and design of the learning experience for students. All articles reported a cross-sectional design (with no mention of further study or follow-up plans), and were broadly aimed at improving learner knowledge, confidence and proficiency in communicating effectively with Deaf and hard of hearing people in a health services setting.

All studies detailed how they developed and designed the Deaf patient simulation, with two studies described as Deaf-led/user-centred showing involvement from initial project stages where there was consultation and data gathering with Deaf communities and Deaf people fully involved in all aspects of the project (Anderson et al., 2020; Blok et al., 2023). Two further studies included one team member with lived experience of deafness as part of the planning and simulation development team, along with other faculty members (McLaughlin et al., 2020; Sanders et al., 2008), but both lacked detail about roles and actual involvement from lived experience team members. The remaining two papers stated the scenarios were developed solely by faculty staff with much less information than the other papers about the simulation and the content (Halley & Connelly, 2022; Yuksel & Unver, 2016). In terms of authenticity, Anderson et al. (2020) and Blok et al. (2023) began projects by collecting data from Deaf communities in focus groups and forums to understand challenges experienced by Deaf people, with resultant content used to develop scripts and videos for simulation.

The types of simulation described in the retrieved studies varied. Simulations with or about Deaf patients included the use of standardised patients played by Deaf individuals in a face-to-face setting (Anderson et al., 2020), or with students acting as standardised patients in a face-to-face setting, watched by other students (Yuksel & Unver, 2016) or a similar arrangement with students taking part as standardised patients on zoom with other students watching virtually (Halley & Connelly, 2022). The remaining three simulations provided a more interactive experience, for example video and questions on a developed CD-ROM which included a filmed scenario (Sanders et al., 2008), a VR experience with an immersive headset and manipulated audio (McLaughlin et al., 2020), and finally computer-based learning using 360 video techniques that could be transferred to headsets, and used without headsets too (Blok et al., 2023).

Topic areas for the simulation included developing a pathway for nursing students to use when communicating with Deaf patients (Yuksel & Unver, 2016) or to effectively gain Deaf individuals’ consent for research (Anderson et al., 2020). Other studies focused on increasing understanding of D/deaf patients’ challenges and experiences in healthcare (Blok et al., 2023), and increasing student competence and confidence in working with Deaf patients (Halley & Connelly, 2022; Sanders et al., 2008), and finally increasing empathy and understanding healthcare experience from Deaf patient perspective (McLaughlin et al., 2020).

Two of the studies adhered to best practice guidelines in simulation education with both pre-brief and de-brief present for learners (Anderson et al., 2020; McLaughlin et al., 2020). The importance of solely de-briefing was highlighted in three further studies (Blok et al., 2023; Halley & Connelly, 2022; Yuksel & Unver, 2016), and not mentioned in the remaining one (Sanders et al., 2008).

With a variety of study topic areas and research aims, the retrieved studies used a range of measures with some identifying a need to develop a specific tool to examine outcomes. Qualitative approaches included exploring what went well, what students learned and what could be improved in the simulation (Halley & Connelly, 2022), as well as student reflections on their experiences of simulation, and their achievements and challenges (Yuksel & Unver, 2016), with others interviewed about their experience, and also asked to submit a rich picture to symbolise their simulation encounter (McLaughlin et al., 2020). In further studies, self-rating and pre and post evaluation measures were used (Anderson et al., 2020), also a system usability survey to explore feasibility of simulation as clinical practice improvement (Blok et al., 2023). One further study team developed a Disability Situations Inventory to measure student comfort level and perceived difficulty (Sanders et al., 2008).

### Outcomes of simulation intervention for participants

The reported outcomes for participants in these simulation education studies in this review included a mixture of quantitative and qualitative measures. All papers included projects that provided a rationale of growing the need for competent and empathic practitioners when working with Deaf and hard of hearing individuals in health service settings. Unsurprisingly researchers who cited participants with ‘very little’ prior experience with Deaf communities reported greater gains in post-intervention performance (Anderson et al., 2020; Sanders et al., 2008).

Practical elements in terms of usability and feasibility of the simulation were measured (Anderson et al., 2020; Blok et al., 2023; Sanders et al., 2008), with mostly positive students’ comments and outcomes. Blok et al. (2023) tested the intervention for system usability with a survey with participants reporting an average of 77.5 for system usability, (where 68 or higher was considered above average); with the study focus on testing feasibility of the intervention. Researchers also stated whether there was a need to prepare participants for the intervention in terms of technical skills, or if technical problems arose, was also highlighted, which on occasion flagged reasons for participants’ non-completion (Blok et al., 2023; Sanders et al., 2008).

Interventions in two studies gave the opportunity for participants to act on feedback from an earlier part of the simulation experience enabling for skill and knowledge development and a ‘second go’ (Anderson et al., 2020; Yuksel & Unver, 2016). Whilst others measured pre and post- simulation intervention for participants’ comfort levels of working with a D/deaf person with significant improvements shown in student comfort level after completing the module (Sanders et al., 2008). Similarly, participants self-reported their confidence levels in communicating with a Deaf person after using the simulation (Anderson et al., 2020; Halley & Connelly, 2022). No standardised measures were used and different studies’ objectives varied with some reporting an increase in the development of their clinical judgement (Halley & Connelly, 2022), and others interested in how the student participants were impacted, in terms of increased empathy (McLaughlin et al., 2020).

As researchers sought to measure the outcomes and impacts of their simulation interventions, various areas were studied. For example, three studies included elements of encouraging reflection in student learning as participants remembered their experiences with deaf people in health care services previously (Yuksel & Unver, 2016) or used self-reflection with critique about the simulation intervention (including the use of reflective journals) (Halley & Connelly, 2022); or reflected by drawing a rich picture to symbolize their intervention experience (McLaughlin et al., 2020), in attempts to get students in touch with their feeling and their learning experiences.

One example of how student groups might strengthen their learning from the simulation intervention was with the development of specific patient pathways for nurses interacting with deaf patients, as demonstrated by Yuksel and Unver (2016). Others made suggestions that they proposed could make the simulation more impactful with participants reporting that the experience would be far more immersive using a headset, than solely a 360 video (Blok et al., 2023). The benefits of immersive elements of simulation intervention were highlighted by Anderson et al., 2020) and Blok et al., (2021), with a focus on reports about increased empathy about what it would be like to be a D/deaf patient in that situation. For example: “Not just what you can’t hear … but how it makes you feel” (McLaughlin et al., 2020 p212, 2020).

Included studies also reported findings from participants about the strengthening of their professional ability and being able to work with others inter-professionally (Halley & Connelly, 2022), noting that first learning together and then working inter-professionally as a team of nurses and interpreters would likely improve healthcare outcomes for Deaf patients.

All authors of included studies acknowledged their small sample sizes and that small exploratory studies had results that may not be transferable, but certainly add value in terms of how simulation interventions might be developed and designed.

## Discussion

To the best of authors’ knowledge there is currently limited evidence about the development and availability of simulation education activities that promote learning about the experiences and communication needs of D/deaf patients. Simulation in health professional education is increasing rapidly due to challenges regarding student placements (Koukourikos et al., 2021^2021^), and advancement in healthcare where immersive technologies can assist students’ learning experience (Komasawa & Yokohira, 2023). However, there are still very few peer-reviewed studies that examine the effectiveness of simulation programmes about the D/deaf patient experience. Very few education providers are focusing on activities that increase student empathy with D/deaf people, and we are aware that where innovations exist, many clinicians and educators focus solely on an audio experience. Providing simulation without audio has value for learners (Llorach et al., 2018) but denies students the opportunity to learn about the wider experiences of being D/deaf, such as barriers to accessing services, and the negative impact on D/deaf people of staff’s limited Deaf awareness.

The review team concurred with Frerejean et al. (2023), that a limited number of simulated educational experiences is not sufficient for students to develop complex skills, as this needs comprehensive programmes that combine simulation sessions with practical and workplace learning. There is no current substitute for students learning in practice with D/deaf patients, so appropriate preparation beforehand, with basic Deaf awareness as a minimum is essential and opportunities for more experiential learning (Terry et al., 2024).

Little is known about how often health professional students encounter D/deaf patients during their training, and only one of the papers in our study asked participants about prior experience with D/deaf individuals (Anderson et al., 2020), with 60% (n = 3) having no experience. With one in five people being D/deaf or hard of hearing (World Health Organization, 2024), we would expect that students would very likely encounter D/deaf patients in practice. However, we know many D/deaf patients avoid healthcare services due to the continual barriers they experience (Wheatley, 2021), which may result in students having few experiences with D/deaf people, and not having those learning opportunities in practice, and so supports the need for simulated learning opportunities.

In the papers included in this review two studies utilised virtual learning approaches (Halley & Connelly, 2022; Sanders et al., 2008), with the potential for training videos/360 experiences described in the other four papers to be utilized in a similar way. According to Birido et al. (2024), plenty of benefits have been found for distance simulation, as delivering simulations remotely may hold advantages of a broader reach to wider geographical locations, access to global experts, potential for increased number of trainees, more simulation sessions, and less time, thereby saving on staffing and resource costs. With little published evidence about the experiences of different learner groups’ simulation experiences about Deafness, it is difficult to know more about specific student populations, but if resource and availability of physical simulation environments is a concern, there is potential for virtual simulation opportunities. Following a large-scale report from PWC (2020), the effectiveness of VR for soft skills is highlighted, and also noted that at scale VR was found to be more cost-effective than classroom based training, in addition to students being more focused, more connected and more confident. The ability to increase remote and “just in time” training opportunities increases efficiency and a more sustainable, flexible, and inclusive way of designing and delivering education across sectors. Kononowicz et al. (2019) systematic review highlighted not only skill and knowledge improvements from the use of VR but also demonstrated the global applicability of this modality.

Low to modest and mixed evidence suggests that when compared with traditional education, virtual patients can more effectively improve skills, and at least as effectively improve knowledge. The skills that improved were clinical reasoning, procedural skills, and a mix of procedural and team skills. We found evidence of effectiveness in both high-income and low- and middle-income countries, demonstrating the global applicability of virtual patients. Further research should explore the utility of different design variants of virtual patients.

The results of our qualitative synthesis suggest potential for further development of learner simulation programmes with the express aim of increasing knowledge and confidence about working with D/deaf patients. We suggest that simulation educators may wish to incorporate a D/deaf patient experience into their suite of immersive or virtual modalities, with many possibilities for creativity that would benefit student learning and may lead to improved communications and increased understanding and empathy with D/deaf patients and families in practice (Llorach et al., 2018). It would be valuable for more rigorous study designs, and for studies to be conducted longitudinally. In terms of enablers, there is sufficient detail in all six papers to inspire and catalyse others to develop similar simulation opportunities. Knowing the importance of embedded lived experience, Anderson et al. (2020) and Blok et al.’s (Blok et al., 2023) studies provide inspiring details about how simulation content was developed by D/deaf communities from the start. We also note the importance of strong inter-disciplinary teams that are required to develop all aspects of a D/deaf patient simulated experience that results in a high quality effective educational opportunity.

### Strengths and limitations

The strengths of our systematic review are the comprehensive search strategy using multiple databases, and the use of explicit in- and exclusion criteria. The PRIMSA guidelines allowed us to develop methods to qualitatively synthesize and report on the various evaluation methods. However, the heterogeneity of the small number of articles that fit the criteria for this study meant that it was not possible to utilize more quantitative synthesis or formal techniques of metaanalysis. While this review provides insight into the delivery of examples of simulated education modalities that focus on learning about D/deaf patients, we made little comment on the rigor of the studies included. All six studies were cross-sectional with small sample sizes and whilst some made claims of significance (Sanders et al., 2008), authors themselves state the need for larger samples and control groups. Although we took a systematic approach to identifying relevant articles, it is possible that we unintentionally overlooked some articles that explored the same phenomenon using different keywords. Due to practical issues, we were only able to include studies in English, which means that some important findings may have been left out. The studies included in our systematic review and our summary do provide exemplars of how to develop a simulation experience to increase knowledge and empathy about D/deaf patients.

## Conclusion

This systematic review suggests there is currently a paucity of evidence about simulation educational experiences that focus on increasing student health professionals’ knowledge and skills about working with D/deaf patients. The team concurred that there is a growing body of evidence supporting the use of simulation and extended reality training methodologies that provide a catalyst for further subject matter specific curriculum development and research. The current research status is too limited to provide educators with evidence-based recommendations on how educators might provide such learning activities but aligning rigorous content, educational and simulation design quality standards with any future works in this area is a recommendation. As retrieved studies were limited in terms of sample size, scope, and study quality, it will be valuable for future research to utilize more rigorous study designs, and to more directly assess the impact of these experiences on clinical practice. Examples of novel projects do exist, and it is hoped that these provide educators, simulation specialists and Deaf communities opportunities for further collaborative work.

## Electronic supplementary material

Below is the link to the electronic supplementary material.

Supplementary Material 1

Supplementary Material 2

Supplementary Material 3

Supplementary Material 4

Supplementary Material 5

Supplementary Material 6

Supplementary Material 7

## Data Availability

No datasets were generated or analysed during the current study.
