# Supplementary material for: Simulated learning interventions to improve communication and practice with deaf and hard of hearing patients: a systematic review and qualitative synthesis
Source: Adv Health Sci Educ Theory Pract. 2025 Jul 9;31(2):495–513. doi: 10.1007/s10459-025-10452-5 (PMC13046636; doi:10.1007/s10459-025-10452-5)
Supplement: Supplementary file 1 — Supplementary Material 1 [file 10459_2025_10452_MOESM1_ESM.pdf]

Search Strategy from ProQuest

15 November 2023 11:40

Supplied by Information Services and Systems, Swansea University.

SEARCH STRATEGY

Set No.	Searched for	Databases	Results
S1	noft(deaf*) OR noft(hard of hearing) OR noft(hearing impaired) OR noft(d/hh) OR noft(d/Deaf)	Applied Social Sciences Index & Abstracts (ASSIA)	2899
S2	noft(sign* language) OR noft(British Sign Language) OR noft(BSL)	Applied Social Sciences Index & Abstracts (ASSIA)	2697
S3	noft(virtual reality) OR noft(VR) OR noft(3D technology) OR noft(simulat*)	Applied Social Sciences Index & Abstracts (ASSIA)	13878
S4	noft(empath*) OR noft(knowledge) OR noft(awareness) OR noft(perspective) OR noft(experience) OR noft(understand*)	Applied Social Sciences Index & Abstracts (ASSIA)	401793
S5	noft([S1]) AND noft([S3]) AND noft([S4])	Applied Social Sciences Index & Abstracts (ASSIA)<br>These databases are searched for part of your query.	14
S6	noft([S2]) AND noft([S3]) AND noft([S4])	Applied Social Sciences Index & Abstracts (ASSIA)<br>These databases are searched for part of your query.	12
S7	noft([S5]) OR noft([S6])	Applied Social Sciences Index & Abstracts (ASSIA)<br>These databases are searched for part of your query.	26
S8	noft(deaf*) OR noft(hard of hearing) OR noft(hearing impaired) OR noft(d/hh) OR noft(d/Deaf)	ProQuest Central	393218
S9	noft(sign* language) OR noft(British Sign Language) OR noft(BSL)	ProQuest Central	238338
S10	noft(virtual reality) OR noft(VR) OR noft(3D technology) OR noft(simulat*)	ProQuest Central	3013550
S11	noft(empath*) OR noft(knowledge) OR noft(awareness) OR noft(perspective) OR noft(experience*) OR noft(understand*)	ProQuest Central	18048682
S12	noft([S8]) AND noft([S10]) AND noft([S11])	ProQuest Central<br>These databases are searched for part of your query.	362
S13	noft([S9]) AND noft([S10]) AND noft([S11])	ProQuest Central<br>These databases are searched for part of your query.	1048
S14	noft([S8]) AND noft([S9]) AND	ProQuest Central<br>These databases are searched for part of your query.	68

	noft([S10]) AND noft([S11])	These databases are searched for part of your query.	
S15	noft([S12]) AND noft([S14])	ProQuest Central<br>These databases are searched for part of your query.	66

Database copyright © 2023 ProQuest LLC. All rights reserved.

Terms and Conditions Contact ProQuest
