# Supplementary material for: Simulated learning interventions to improve communication and practice with deaf and hard of hearing patients: a systematic review and qualitative synthesis
Source: Adv Health Sci Educ Theory Pract. 2025 Jul 9;31(2):495–513. doi: 10.1007/s10459-025-10452-5 (PMC13046636; doi:10.1007/s10459-025-10452-5)
Supplement: Supplementary file 2 — Supplementary Material 2 [file 10459_2025_10452_MOESM2_ESM.pdf]

Thursday, November 16, 2023 4:34:49 PM

#	Query	Limiters/Expanders	Last Run Via	Results
S7	S5 OR S6	Expanders - Apply equivalent subjects<br>Search modes - Boolean/Phrase	Interface - EBSCOhost Research Databases<br>Search Screen - Advanced Search<br>Database - CINAHL Plus with Full Text	193
S6	S1 AND S3 AND S4	Expanders - Apply equivalent subjects<br>Search modes - Boolean/Phrase	Interface - EBSCOhost Research Databases<br>Search Screen - Advanced Search<br>Database - CINAHL Plus with Full Text	193
S5	S1 AND S2 AND S3 AND S4	Expanders - Apply equivalent subjects<br>Search modes - Boolean/Phrase	Interface - EBSCOhost Research Databases<br>Search Screen - Advanced Search<br>Database - CINAHL Plus with Full Text	5
S4	empath* OR knowledge OR awareness OR perspective OR experience* OR understand*	Expanders - Apply equivalent subjects<br>Search modes - Boolean/Phrase	Interface - EBSCOhost Research Databases<br>Search Screen - Advanced Search<br>Database - CINAHL Plus with Full Text	1,251,248
S3	virtual reality OR VR OR 3d technology OR simulat*	Expanders - Apply equivalent subjects<br>Search modes - Boolean/Phrase	Interface - EBSCOhost Research Databases<br>Search Screen - Advanced Search<br>Database - CINAHL Plus with Full Text	98,797
S2	sign* language OR British Sign Language OR BSL	Expanders - Apply equivalent subjects<br>Search modes - Boolean/Phrase	Interface - EBSCOhost Research Databases<br>Search Screen - Advanced Search<br>Database - CINAHL Plus with Full Text	5,449

S1	deaf* OR hard of hearing OR hearing impaired OR d/hh OR d/Deaf	Expanders - Apply equivalent subjects<br>Search modes - Boolean/Phrase	Interface - EBSCOhost Research Databases<br>Search Screen - Advanced Search<br>Database - CINAHL Plus with Full Text	55,482
