# Supplementary material for: Simulated learning interventions to improve communication and practice with deaf and hard of hearing patients: a systematic review and qualitative synthesis
Source: Adv Health Sci Educ Theory Pract. 2025 Jul 9;31(2):495–513. doi: 10.1007/s10459-025-10452-5 (PMC13046636; doi:10.1007/s10459-025-10452-5)
Supplement: Supplementary file 4 — Supplementary Material 4 [file 10459_2025_10452_MOESM4_ESM.pdf]

Friday, November 17, 2023 9:14:11 AM

| # | Query | Limiters/Expanders | Last Run Via | Results |
|---|---|---|---|---|
| S11 | S6 OR S7 OR S8 | Limiters - Peer Reviewed<br>Expanders - Apply equivalent subjects<br>Narrow by Language: - english<br>Search modes - Boolean/Phrase | Interface - EBSCOhost Research Databases<br>Search Screen - Advanced Search<br>Database - MEDLINE | 466 |
| S10 | S6 OR S7 OR S8 | Limiters - Peer Reviewed<br>Expanders - Apply equivalent subjects<br>Search modes - Boolean/Phrase | Interface - EBSCOhost Research Databases<br>Search Screen - Advanced Search<br>Database - MEDLINE | 480 |
| S9 | S6 OR S7 OR S8 | Expanders - Apply equivalent subjects<br>Search modes - Boolean/Phrase | Interface - EBSCOhost Research Databases<br>Search Screen - Advanced Search<br>Database - MEDLINE | 486 |
| S8 | S3 AND S4 AND S5 | Expanders - Apply equivalent subjects<br>Search modes - Boolean/Phrase | Interface - EBSCOhost Research Databases<br>Search Screen - Advanced Search<br>Database - MEDLINE | 385 |
| S7 | S1 AND S3 AND S4 | Expanders - Apply equivalent subjects<br>Search modes - Boolean/Phrase | Interface - EBSCOhost Research Databases<br>Search Screen - Advanced Search<br>Database - MEDLINE | 390 |
| S6 | S1 AND S2 AND S3 AND S4 | Expanders - Apply equivalent subjects<br>Search modes - Boolean/Phrase | Interface - EBSCOhost Research Databases<br>Search Screen - Advanced Search<br>Database - MEDLINE | 9 |
| S5 | (MH "Deafness+") OR (MH "Hearing Loss+") OR (MH "Usher Syndromes") OR "deaf" | Expanders - Apply equivalent subjects<br>Search modes - Boolean/Phrase | Interface - EBSCOhost Research Databases<br>Search Screen - Advanced Search<br>Database - MEDLINE | 119,830 |

| | | | | |
|---|---|---|---|---|
| S4 | empath* OR knowledge OR awareness OR perspective OR experience* OR understand* | Expanders - Apply equivalent subjects<br>Search modes - Boolean/Phrase | Interface - EBSCOhost Research Databases<br>Search Screen - Advanced Search<br>Database - MEDLINE | 4,171,706 |
| S3 | virtual reality OR VR OR 3d technology OR simulat* | Expanders - Apply equivalent subjects<br>Search modes - Boolean/Phrase | Interface - EBSCOhost Research Databases<br>Search Screen - Advanced Search<br>Database - MEDLINE | 939,334 |
| S2 | sign* language OR British Sign Language OR BSL | Expanders - Apply equivalent subjects<br>Search modes - Boolean/Phrase | Interface - EBSCOhost Research Databases<br>Search Screen - Advanced Search<br>Database - MEDLINE | 10,329 |
| S1 | deaf* OR hard of hearing OR hearing impaired OR d/hh OR d/Deaf | Expanders - Apply equivalent subjects<br>Search modes - Boolean/Phrase | Interface - EBSCOhost Research Databases<br>Search Screen - Advanced Search<br>Database - MEDLINE | 108,969 |
