# Supplementary figures and images for: Simulated learning interventions to improve communication and practice with deaf and hard of hearing patients: a systematic review and qualitative synthesis
Source: Adv Health Sci Educ Theory Pract. 2025 Jul 9;31(2):495–513. doi: 10.1007/s10459-025-10452-5 (PMC13046636; doi:10.1007/s10459-025-10452-5)

Scopus 15/11/2023


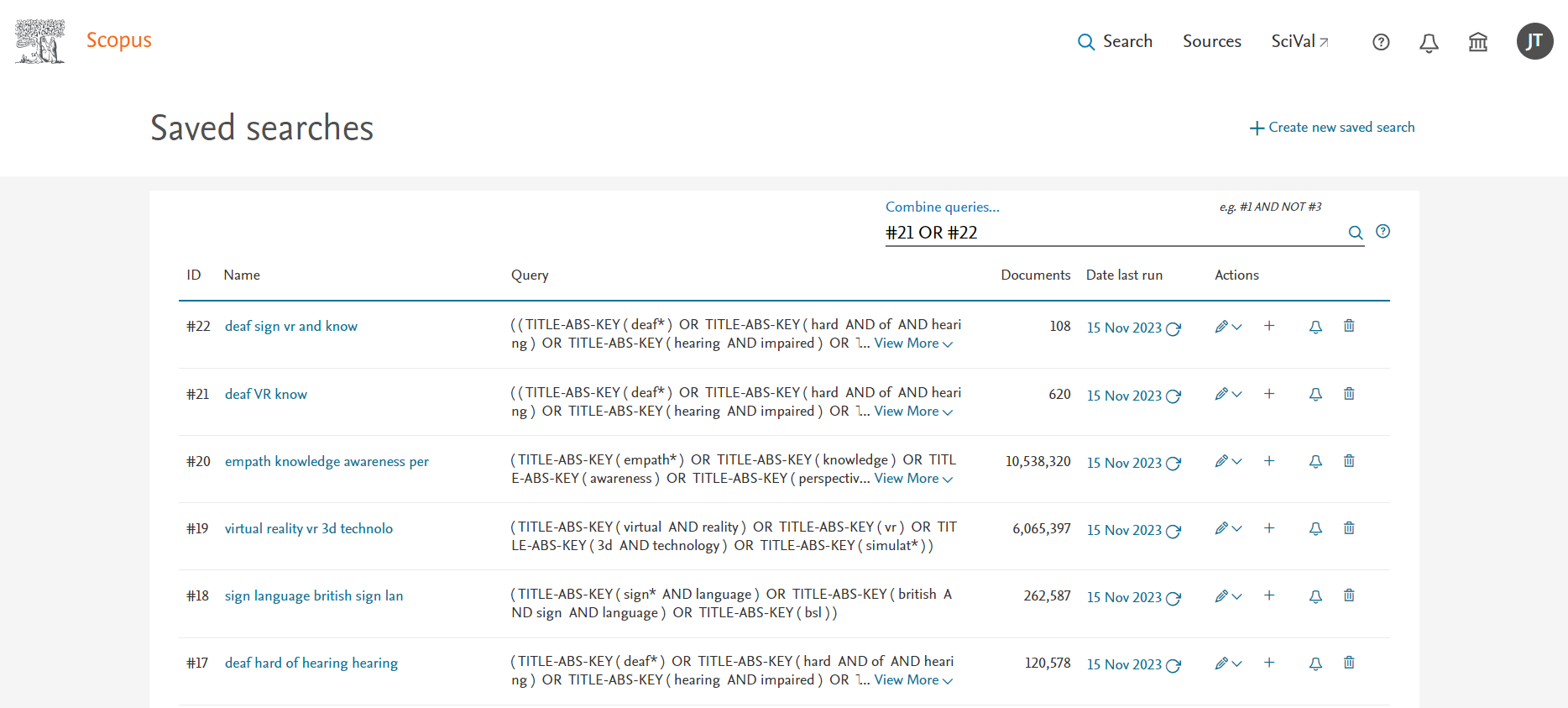

Supplement: Supplementary file 5 — Supplementary Material 5 [file 10459_2025_10452_MOESM5_ESM.docx]
