# Supplementary material for: Simulated learning interventions to improve communication and practice with deaf and hard of hearing patients: a systematic review and qualitative synthesis
Source: Adv Health Sci Educ Theory Pract. 2025 Jul 9;31(2):495–513. doi: 10.1007/s10459-025-10452-5 (PMC13046636; doi:10.1007/s10459-025-10452-5)
Supplement: Supplementary file 7 — Supplementary Material 7 [file 10459_2025_10452_MOESM7_ESM.pdf]

Web of Science Search Strategy (v0.1)

Search: deaf* (Topic) OR hard of hearing (Topic) OR hearing impaired (Topic) OR d/hh (Topic) OR d/Deaf (Topic) Date Run: Wed Nov 15 2023 12:20:28 GMT+0000 (Greenwich Mean Time) Results: 62490

Search: virtual reality (Topic) OR VR (Topic) OR 3D technology (Topic) OR simulat* (Topic) Date Run: Wed Nov 15 2023 12:31:28 GMT+0000 (Greenwich Mean Time) Results: 4311104

Search: empath* (Topic) OR knowledge (Topic) OR awareness (Topic) OR perspective (Topic) OR experience* (Topic) OR understand* (Topic) Date Run: Wed Nov 15 2023 12:32:34 GMT+0000 (Greenwich Mean Time) Results: 7821040

Search: #3 AND #2 AND #1 Date Run: Wed Nov 15 2023 12:33:39 GMT+0000 (Greenwich Mean Time) Results: 383

Database: Web of Science Core Collection

Entitlements:

- WOS.SCI: 1970 to 2023
- WOS.AHCI: 1975 to 2023
- WOS.ESCI: 2015 to 2023
- WOS.ISTP: 1990 to 2023
- WOS.SSCI: 1970 to 2023
- WOS.ISSHP: 1990 to 2023

Searches:

Search: #3 AND #2 AND #1 and Article (Document Types) and English (Languages) Date Run: Wed Nov 15 2023 12:36:02 GMT+0000 (Greenwich Mean Time) Results: 274
